# Escore SAGE e Velocidade da Onda de Pulso em Indivíduos sem Hipertensão Arterial

**DOI:** 10.36660/abc.20220881

**Published:** 2023-02-13

**Authors:** Alfredo José Mansur

**Affiliations:** 1 Instituto do Coração Faculdade de Medicina Universidade de São Paulo São Paulo SP Brasil Instituto do Coração da Faculdade de Medicina da Universidade de São Paulo, São Paulo, SP – Brasil

**Keywords:** Hipertensão, Análise da Onda de Pulso/métodos/tendências, Ritmo Circadiano, Artérias Carótidas, Rigidez Vascular, Envelhecimento/fisiologia, Fatores de Risco, Estresse Oxidativo, Calcificação Vascular

Uma das etapas do exame clínico cardiológico é o exame dos pulsos arteriais. Na inspeção, na palpação e na ausculta identificamos características semiológicas do pulso arterial – a simetria dos pulsos nos dimídios direito e esquerdo, o ritmo, a regularidade, a frequência, a rigidez das paredes arteriais (particularmente artérias carótidas), a pressão de pulso e a velocidade de ascensão da onda de pulso e a presença de frêmitos e sopros.^1^ Mais recentemente a tecnologia desenvolveu-se de modo a permitir a avaliação, o registro e a possibilidade de observações evolutivas da rigidez arterial, por meio do estudo pressão arterial periférica e central, pressão de pulso periférica e central, da velocidade da onda de pulso e do índice de amplificação ( *augmentation index* ).^2 , 3^ O aumento da rigidez arterial se exprime por aumento da velocidade da onda de pulso^4^ e a interação entre a velocidade da onda de pulso a jusante e a onda refletida exprime-se no índice de amplificação.^4^ As alterações estruturais e funcionais da vasculatura poderiam explicar as alterações relacionadas à senescência e constituírem per se um fator de risco cardiovascular.^5^ Estudadas em amostras de portadores de hipertensão arterial,^6^ o estudo da rigidez arterial é também de interesse em indivíduos não hipertensos.

No estudo^7^ os pesquisadores avaliaram o quanto indicadores clínicos (idade e pressão arterial sistólica periférica) e metabólicos (glicemia, taxa estimada de filtração glomerular) reunidos em uma escore (SAGE)^8^ se relacionariam com variáveis medidas nas estimativas de rigidez arterial avaliadas por método não invasivo em 100 participantes normotensos (n= 80) ou pré-hipertensos (n=20) de uma amostra de 1594 pacientes; o diagnóstico de hipertensão arterial (n=1349) foi um critério de exclusão na análise. A pontuação máxima do escore empregado é 17. A pontuação mais frequente observada na amostra ficou entre 0 e 3 (64% da amostra). A mediana da velocidade da onda de pulso nessa amostra foi 7,3 m.seg^-1^. À medida que se aumentou o ponto de corte da velocidade da onda de pulso (percentis 50% - 7,3 m.seg^-1^ e 75% - 9,8 m.seg^-1^) elevou os valores preditivos positivos e negativos da identificação da rigidez arterial com base no aumento dos escores SAGE registrados na amostra de estudo. Nos participantes com velocidade da onda de pulso superior a 10 m.seg ^-1^ o escore mais frequente foi 7. Os autores concluíram que o escore SAGE permitiria identificar participantes não hipertensos com maior frequência de rigidez arterial. Isto posto, estudos adicionais poderiam identificar o benefício de intervenções nessa população para prevenir hipertensão arterial ou outras afecções cardíacas ou vasculares, além de permitir estudos evolutivos, garantida a reprodutibilidade das medidas.

O estudo do envelhecimento arterial com a contribuição da possibilidade de registro não invasivo também toma em consideração os mecanismos estruturais,^5^ subcelulares e moleculares^9 , 10^ que participam da tradução hemodinâmica para as modificações nas paredes arteriais. Há estudos feitos em diferentes populações que avaliaram as velocidades de onda de pulso registradas de tal forma que pudessem indicar velocidade mais altas e que significariam maior risco cardiovascular.^11^ Admite-se que a artéria senescente se caracteriza por disfunção endotelial, inflamação crônica, estresse oxidativo, migração e proliferação de células musculares lisas e calcificação arterial.^12^ Outro mecanismo aventado para a calcificação arterial foi a exposição a fosfato extracelular e tissular, nos quais pode se precipitar o cálcio e iniciar o processo de calcificação.^13^ Eventualmente outros métodos de avaliação podem também vir a contribuir com esse conhecimento como a ressonância magnética.^14^

O estudo da rigidez arterial é campo de investigação de interesse e que deve trazer novos conhecimentos, tendo em vista a sugestão de que o envelhecimento vascular possa não ser considerado um evento inexorável mas passível de intervenções terapêuticas específicas no futuro,^12^ além das medidas tradicionais, entre eles a idade, a dieta saudável, atividade física regular,^15^ a obesidade, o tabagismo, a hipercolesterolemia, a hipertensão arterial, o diabetes mellitus.^16 , 17^

## References

[1] Walker HK, Hall WD, Hurst JW (1980). Clinical methods. The hystory, physical and laboratory examinations.

[2] Safar ME (2010). Arterial aging--hemodynamic changes and therapeutic options. Nat Rev Cardiol.

[3] McEniery CM, Cockcroft JR, Roman MJ, Franklin SS, Wilkinson IB (2014). Central blood pressure: current evidence and clinical importance. Eur Heart J.

[4] Lee HY, Oh BH (2010). Aging and arterial stiffness. Circ J.

[5] Thijssen DH, Carter SE, Green DJ (2016). Arterial structure and function in vascular ageing: are you as old as your arteries?. J Physiol.

[6] Sharman JE, Laurent S (2013). Central blood pressure in the management of hypertension: soon reaching the goal?. J Hum Hypertens.

[7] Rigonatto RRF, Vitorino PVO, Oliveira AC, Sousa ALL, Jardim PCBV, Cunha PMGM (2023). SAGE Score in Normotensive and Pre-Hypertensive Patients: A Proof of Concept. Arq Bras Cardiol.

[8] Xaplanteris P, Vlachopoulos C, Protogerou AD, Aznaouridis K, Terentes-Printzios D, Argyris AA (2019). A clinical score for prediction of elevated aortic stiffness: derivation and validation in 3943 hypertensive patients. J Hypertens.

[9] Ungvari Z, Tarantini S, Donato AJ, Galvan V, Csiszar A (2018). Mechanisms of Vascular Aging. Circ Res.

[10] Wang M, Monticone RE, McGraw KR (2018). Proinflammatory Arterial Stiffness Syndrome: A Signature of Large Arterial Aging. J Vasc Res.

[11] Kim HL, Kim SH (2019). Pulse Wave Velocity in Atherosclerosis. Front Cardiovasc Med.

[12] Tesauro M, Mauriello A, Rovella V, Annicchiarico-Petruzzelli M, Cardillo C, Melino G (2017). Arterial ageing: from endothelial dysfunction to vascular calcification. J Intern Med.

[13] Bäck M, Michel JB (2021). From organic and inorganic phosphates to valvular and vascular calcifications. Cardiovasc Res.

[14] Ohyama Y, Redheuil A, Kachenoura N, Venkatesh BA, Lima JA (2018). Imaging Insights on the Aorta in Aging. Circ Cardiovasc Imaging.

[15] Kresnajati S, Lin YY, Mündel T, Bernard JR, Lin HF, Liao YH (2022). Changes in Arterial Stiffness in Response to Various Types of Exercise Modalities: A Narrative Review on Physiological and Endothelial Senescence Perspectives. Cells.

[16] Teo KK, Rafiq T (2021). Cardiovascular Risk Factors and Prevention: A Perspective From Developing Countries. Can J Cardiol.

[17] Force US Preventive Services Task, Mangione CM, Barry MJ, Nicholson WK, Cabana M, Coker TR, Davidson KW (2022). Behavioral Counseling Interventions to Promote a Healthy Diet and Physical Activity for Cardiovascular Disease Prevention in Adults Without Cardiovascular Disease Risk Factors: US Preventive Services Task Force Recommendation Statement. JAMA.

