# Supplementary material for: Escore SAGE e Velocidade da Onda de Pulso em Indivíduos sem Hipertensão Arterial
Source: Arq Bras Cardiol. 2023 Feb 13;120(2):e20220881. [Article in Portuguese] doi: 10.36660/abc.20220881 (PMC10263455; doi:10.36660/abc.20220881)
Supplement: Translation

# Translation: SAGE Score and Pulse Wave Velocity in Non-Hypertensive Individuals

**Authors:** Alfredo José Mansur

**Affiliations:** 1 Brasil Instituto do Coração da Faculdade de Medicina da Universidade de São Paulo, São Paulo, SP – Brasil

**Keywords:** Hypertension, Pulse Wave Analysis/methods, trends, Circadian Rhythm, Carotid Arteries, Vascular Stiffness, Aging/physiology, Risk Factors, Oxidative Stress, Vascular Calcification

An important step of physical examination is the evaluation of arterial pulses. On inspection, palpation and auscultation we are able to study the characteristics of the arterial pulses – symmetry, rate, rhythm, rigidity of arterial wall (mainly in carotid arteries), pulse pressure, contour, velocity of ascension of the ulse wave, thrill, bruits and murmurs.^1^Current day technology made possible to register graphically the pulse wave and to make serial evaluations of arterial rigidity by studying central and peripheral blood pressure, central and peripheral pulse pressure, pulse wave velocity e augmentation index.^2 , 3^An increase in arterial rigidity leads to increase in the pulse wave velocity^4^and the interaction between the forward pulse wave with the backward reflexion is observed in the augmentation index.^4^Anatomical and functional changes of the arteries is one of the mechanisms involved in senescence and may constitute per se a cardiovascular risk factor.^5^Studies of arterial rigidity were performed in patients with arterial hypertension.^6^Indeed, the study of arterial rigidity is also interesting in samples of individuals without hypertension.

In the study^7^researchers reported a study devised to evaluate clinical (age, peripheral systolic arterial pressure) and metabolic (serum glucose, estimated glomerular filtration rate) characteristics in a previously published score (SAGE)^8^relative to variables of arterial rigidity evaluated non-invasively in a sample of 100 participants with normal blood pressure (n=80) or pre-hypertensive (n=20) out of a large sample of 1594 patients; diagnosis of arterial hypertension (n=1349) was an exclusion criteria for this analysis. The maximal score value is 17. The distribution of the score in this study sample ranged between 0 and 3 (64% of the sample). Median of pulse wave velocity in this study sample was 7,3 m.seg^-1^. The increase in cut off value of pulse wave velocity (percentis 50% - 7,3 m.seg^-1^e 75% - 9,8 m.seg^-1^) was associated with an increase in positive and negative predictive values on the basis of scores SAGE observed in the study sample. In participants with pulse wave velocity higher than 10 m.seg^-1^the most frequent score was 7. The authors concluded that the SAGE score might be useful to screening non hypertensive subjects with higher risk of increase in arterial rigidity. Additional studies might be of help of therapeutic interventions in these clinical scenarios, including follow-up studies provided appropriate reproducibility of the measurements.

The study of arterial senescence with the help of non-invasive registry of pulse wave velocity also take involve structural^5^as well as subcellular and molecular mechanisms^9 , 10^active in the hemodynamic consequences of changes in arterial wall. Researches performed in different populations evaluated values of pulse wave velocity that might identify higher cardiovascular risk.^11^Senecescent arteries may be associated with endothelial dysfunction, chronic inflammation, oxidative stress, migration and proliferation of smooth muscle cells and calcification.^12^Other mechanism suggested for arterial calcification is the exposition to extracellular and tissular phosphates favouring calcium deposition and beginning of calcification.^13^New methods such as magnetic resonance may also add to the study of arterial rigidity.^14^

In closing, the study of arterial rigidity is an active research field and new knowledge may be of therapeutic value, in such a way that vascular senescence might not be considered inexorable but an opportunity for prevention and specific therapy in the future^12^in addition to traditional risk factors such as age, healthy diet, exercise,^15^obesity, smoking, hypercholesterolemia, hypertension and diabetes.^16 , 17^
